# Acute Contractile Effects of Glucagon-like-Peptide-1 Receptor Agonists in the Human Heart

**DOI:** 10.3390/pharmaceutics18040447

**Published:** 2026-04-06

**Authors:** Joachim Neumann, Uwe Kirchhefer, Britt Hofmann, Ulrich Gergs

**Affiliations:** 1 Institute for Pharmacology and Toxicology, Medical Faculty, Martin-Luther University Halle-Wittenberg, Magdeburger Straße 4, D-06097 Halle (Saale), Germany; ulrich.gergs@medizin.uni-halle.de; 2 Institute for Pharmacology and Toxicology, Medical Faculty, University Münster, Domagkstraße 12, D-48149 Muenster, Germany; kirchhef@medizin.uni-muenster.de; 3 Department of Cardiac Surgery, Mid-German Heart Centre, University Hospital Halle, Ernst-Grube Straße 40, D-06097 Halle (Saale), Germany; britt.hofmann@uk-halle.de

**Keywords:** Glucagon-like peptide receptor agonists, semaglutide, orforglipron

## Abstract

Glucagon-like-peptide-1 receptor (GLP-1R) agonists are under development as new drugs to treat type 2 diabetes, liver disease, obesity and cardiovascular diseases. Some of these drugs are solely agonists of the GLP-1R. It turned out that their benefit could be improved when they also stimulated the glucagon receptor (GCGR) and/or the glucose-dependent insulinotropic polypeptide (GIP) receptor (GIPR). Stimulation of GLP-1R in cell cultures but also in neonatal atrial and/or ventricular cardiomyocytes and adult atrial cardiomyocytes raised the activity of adenylyl cyclase and thus augmented the 3’,5’cyclic adenosine monophosphate (cAMP) levels. We discuss here the acute contractile effects of such agonists on isolated human atrial and ventricular cardiac preparations from failing and non-failing hearts. We address the receptors involved, GLP-1R expression in various cardiac regions of the human heart, single and multiple receptor agonists and the post-receptor signal transduction system of the GLP-1R in the human heart. Some of the new drugs addressed are still in the early phases of clinical development. We critically discuss the experimental and clinical data available and we also define research needs for experimental and clinical studies.

## 1. Introduction

Glucagon-like-peptide-1 (GLP-1) induces secretion of insulin in the endocrine pancreas [1]. GLP-1 itself was identified when trying to understand the so-called incretin effect. The incretin effect means, in mammals, infusion of glucose into veins releases less insulin from the pancreas than per oral application of the same amount of glucose [2]. Hence, researchers looked for peptides that are produced in the gut after oral intake of glucose and that release insulin from the pancreas [1,2]. Apparently, Walter Creutzfeld in Göttingen, Germany [3,4], Joel Francis Habener [5] in Boston, MA, USA and Jens Juul Holst [2] Copenhagen, Denmark were the first to identify this peptide as GLP-1 [1,6,7,8,9]. GLP-1 has a multitude of effects: GLP-1 may improve glycemic control, inhibit gastric emptying, reduce appetite, impair pancreatic β-cell apoptosis, and at least in animal experiments may lead to pancreatic β-cell proliferation, may regulate gut motility and may reduce inflammation [1]. GLP-1 leads to satiety and this reduces food intake [2,8,10]. Moreover, GLP-1 and by extension GLP-1R agonists may improve memory, may have antidepressant effects, may enhance learning, and may have cardioprotective roles and may have general neuroprotective roles [9,11,12].

The development of clinically useful GLP-1R agonists started by serendipity: in the saliva of certain lizards, several peptides were identified that could decrease blood glucose levels in experimental animals. One of these peptides was named exendin-4 [13]. Exendin-4 was synthesized and got drug approval under the name “exenatide” [14] (Table 1 and Table 2). Exendin-4-containing extracts (from the lizard saliva) heightened adenylyl cyclase activity in both pancreatic preparations and in stomach chief cells from guinea pigs [13]. This was the first evidence that GLP-1R elevated the activity of adenylyl cyclases in mammals. Research on GLP-1R gained a firm ground when the human GLP-1R was cloned [15,16]. Exendin-4 bound to the same receptor (GLP-1R) as GLP-1 [13]. Exendin-4 is more resistant to proteases than GLP-1. Indeed, native GLP-1 is so rapidly cleaved by proteases that the half-life of GLP-1 amounts to a few minutes. This short half-life of GLP-1 is due to the rapid degradation of GLP-1 by dipeptidyl peptidase-4 (DPP-4) or to the neutral endopeptidase 24.11 and, to a lesser extent, to renal elimination [17]. Therefore, a constant theme in the compounds listed below (Table 2 and Table 3) was the necessity to devise molecules with longer half-life than GLP-1. There have been attempts to prolong the half-life in such a way that a GLP-1R agonist can be injected only once a week or once a month. In the present context, it seems relevant that GLP-1R agonists are not only used to reduce elevated glucose levels in patients with type 2 diabetes. In addition, clinical studies indicated that some GLP-1R agonists have cardioprotective effects (Table 1). Hence, GLP-1R agonists seem to exert direct and/or indirect beneficial effects in the human heart. We start the present review with general studies on GLP-1 and GLP-1R. Next, we will address selective GLP-1R agonists in chronological order. Several GLP-1R agonists are already on the market and a few are predicted to enter the market in the near future (Table 1). It is noteworthy that currently the interest seems to be shifting from drugs that only stimulate the GLP-1R to more novel drugs that also act on other receptors which we will therefore address subsequently. Some of these additional receptors like the glucagon receptors (GCGRs) or the glucose-dependent insulinotropic polypeptide (GIPR) also occur in the heart and exert positive inotropic effects on their own in the isolated human atrium [18,19,20]. Especially the new dually or triple agonistic drugs are in our eyes promising and might lead to more effective drugs and are therefore also addressed in this review. For rapid reference, the drugs in the subsequent sections are shown in alphabetical order in the second column of Table 2 (for drugs that solely act on the GLP-1R) and of Table 3 (for drugs that also act on other receptors).

## 2. Signal Transduction

**Figure 1:**
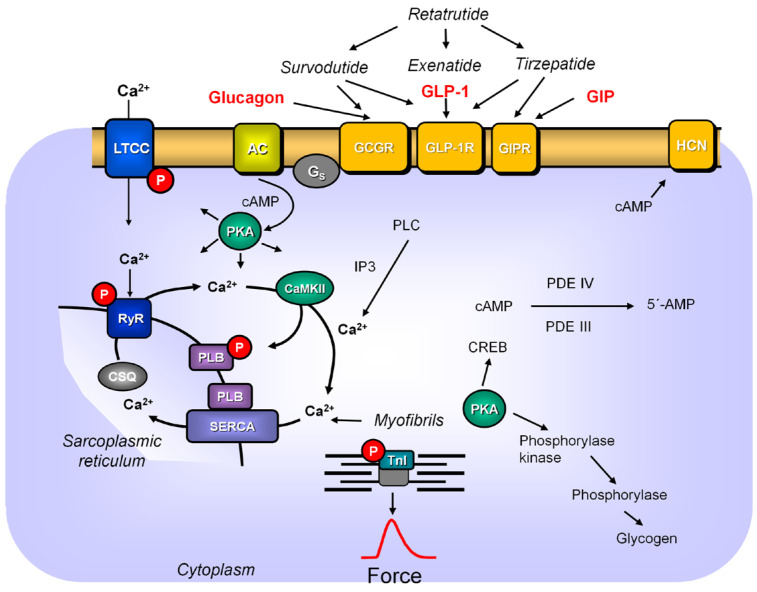
Schematic action of selective or non-selective GLP-1R agonists. Glucagon, GLP-1 and GIP can activate the glucagon receptor (GCGR), the glucagon-like-protein 1 receptor (GLP-1R) and the glucose-dependent insulinotropic polypeptide (GIPR) in cardiomyocytes, respectively. Then, the stimulatory GTP-binding protein (Gs) can activate adenylyl cyclase (AC) and thereafter cAMP is formed. This cAMP activates the cAMP-dependent protein kinase (PKA). This PKA phosphorylates and activates phospholamban (PLB) and the ryanodine receptor (RYR), both located in the sarcoplasmic reticulum, as well as the inhibitory subunit of troponin (TnI) in the myofilaments, the L-type calcium ion channels (LTCC) in the sarcolemma and the cAMP-responsive-element-binding protein (CREB) in the nucleus. PKA can also phosphorylate phosphorylase kinase, which phosphorylates and activates phosphorylase and this leads to cleavage of glycogen. GCGR, GLP-1R and GIPR can probably also activate phospholipase C (PLC). PLC then forms inositoltrisphosphate (IP3) which can release Ca^2+^ from in the sarcoplasmic reticulum. Phospholamban in its unphophorylated form inhibits the activity of the sarcoplasmic reticulum Ca^2+^ ATPase (SERCA) which pumps Ca^2+^ from the cytosol into the sarcoplasmic reticulum. Phosphorylated phospholamban is less inhibitory on SERCA and this facilitates the uptake of Ca^2+^ into the sarcoplasmic reticulum from the cytosol. This leads to faster mechanical relaxation of the heart. Ca^2+^ is stored mainly by calsequestrin (SR) in the sarcoplasmic reticulum. Upon phosphorylation RYR releases Ca^2+^ from the sarcoplasmic reticulum which can act on the myofilaments to raise force of contraction faster. The opening of the RYR is probably also potentiated by Ca^2+^ which enters the cardiomyocyte via the LTCC. The cAMP may directly activate the hyperpolarization-activated cyclic nucleotide-gated channels (HCN). This leads to depolarization and can explain why cAMP-increasing drugs can lead to tachycardia. The formed cAMP in the heart is degraded to inactive 5’-AMP in the mouse heart or the human heart mainly by phosphodiesterase (PDE) 3 and 4, respectively. Released Ca^2+^ from the sarcoplasmic reticulum can activate the calmodulin-dependent protein kinase II (CaMKII). CaMKII phosphorylates and activates PLB. As typical drugs, we indicate that exenatide selectively activates the GLP-1R like some other clinically used drugs (Table 2). In contrast, survodutide and many other compounds (Table 3) in this review are dual activators of GLP-1R and of GCGR. Tirzepatide like some similar drugs activates the GLP-1R and also the GIPR (Table 3). Finally, retatrutide like some similar drugs stimulates all three mentioned receptors.

**Figure 2:**
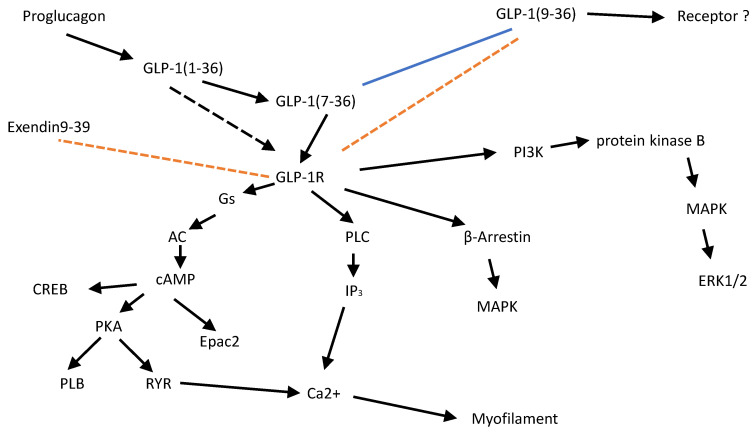
Signal transduction in HAP. Proglucagon is proteolyzed to GLP-1(1-36). GLP-1(1-36) can be truncated to the active metabolite GLP-1(7-36). GLP-1(1-36) and much more potently GLP-1(7-36) can activate the glucagon-like-protein 1 receptor (GLP-1R). Then, at least three different signal pathways can be used: the stimulatory GTP-binding protein (Gs) can activate adenylyl cyclase (AC) and thereafter cAMP is formed. As also seen in Figure 1, this cAMP activates the cAMP-dependent protein kinase (PKA). This PKA phosphorylates and activates phospholamban (PLB) and the ryanodine receptor (RYR), both located in the sarcoplasmic reticulum and cAMP-responsive-element-binding protein (CREB) in the nucleus. In addition, cAMP can activate the Epac2. GLP-1R can also activate phospholipase C (PLC). PLC then forms inositoltrisphosphate (IP3) which can release Ca^2+^ from internal stores. In addition, PLC may activate protein kinase C (PKC) which may open L-type calcium ion channels (LTCCs) via transient receptor potential channel member 4 (TRPM4) and TRPM5. Then increased Ca^2+^ can act on the myofilaments to raise force of contraction. GLP-1R can also use a non-canonical pathway to activate β-arrestin and thereafter the mitogen-activated protein kinase (MAPK). GLP-1R can also activate phosphatidylinositol 3-kinases (PI3Ks) which then activate protein kinase B, then MAPK and extracellular-regulated kinases (ERK1/2). From GLP-1 isoforms, a protease can cleave two amino acids to form GLP-1(9-36) which is an antagonist of GLP-1R. GLP-1(9-36) might activate a putative, unknown receptor.

Animal studies: The signal transduction of the GLP-1R in pancreatic cells involves 3’,5’cyclic adenosine monophosphate (cAMP, Figure 1 and Figure 2), cAMP-dependent protein kinase, but also cAMP-regulated guanine nucleotide exchange factors (Epac), mitogen activated protein kinase (MAPK), phosphoinositide kinase 3 (PI-3K), protein kinase C, phospholipase C, L-type calcium ion channels (LTCCs), ryanodine receptors and Ca^2+^ mobilization [57]. In principle, these biochemical steps are probably also employed in human cardiomyocytes. It has been suggested that dual or triple agonists are superior to pure GLP-1R agonists because of their complementary or synergistic nature to activate several receptor systems in organs that control body weight [58]. That can mean that they act on a certain receptor in the pancreas (e.g., the GLP-1R) and the same or a synergistic receptor in the brain (e.g., glucose-dependent insulinotropic polypeptide: GIPR, glucagon receptor: GCGR, Figure 1). However, there is evidence that they can also act on different cell types in a single organ such as the pancreas and in all likelihood in the heart. They may even act synergistically on a single cell type, in our case, cardiomyocytes (GLP-1, GCGR, GIPR). The GLP-1R couples in human cardiomyocytes via stimulatory guanosine-triphosphate-binding proteins (G-proteins) to activate adenylyl cyclase and to induce cAMP production (Figure 1 and Figure 2 [59]). GLP-1R couples to some extent with other G-proteins [59]. Data in non-cardiac tissue suggest that only high concentrations of GLP-1R agonists (nanomolar concentrations) use cAMP for their functional effects. In contrast, lower, physiological (picomolar) concentrations of GLP-1 appear to work via PLC-mediated pathways [60] acting by protein kinase C via inositol-triphosphate-induced Ca^2+^ release and the activation of the L-type calcium ion channel in non-cardiac cells [61]. It remains to be elucidated whether picomolar concentrations of GLP-1 in the animal and human heart also use these pathways. Furthermore, one may discern acute from chronic effects of GLP-1R stimulation. Here, we mainly address acute effects of GLP-1R agonists. However, clinically long-term effects are also relevant. For instance, long-term treatment (i.e., one week of injection with the GLP-1R agonist liraglutide) enhanced the activity of adenosine-mono-phosphate kinase, glycogen-synthase-kinase3β and ERK in adult wild-type mouse hearts [62].

Another relevant observation is that transfected GLP-1R showed rapid desensitization in cell culture work [63]. From this observation follows another way to classify and potentially improve on GLP-1R agents: namely, whether they are biased or non-biased agonists. GLP-1R may use the non-canonical pathway via β-arrestin and extracellular signal-regulated kinase (ERK) activation [1,64]. The concept behind this approach is that biased agonists might be better suited to reduce body weight than non-biased drugs. On the one hand, if a GLP-1R agonist induces rapid desensitization by the help of β-arrestin then this drug will act only briefly and should be ineffective to reduce body weight in the long run [65,66]. On the other hand, a GLP-1R agonist that does not induce desensitization should exert a more prolonged body weight reduction.

Human studies: The positive inotropic effects of the GLP-1R agonist exenatide (cf. Section 6) in human atrial preparations were accompanied by enhanced phosphorylation of phospholamban at its amino acid serine 16 (Figure 1). This is the phosphorylation site of the cAMP-dependent protein kinase in phospholamban and this supports the view that GLP-1R agonists act, at least in part, via cAMP in the human heart. The rise in force due to exenatide was diminished by an inhibitor of cAMP-dependent protein kinase (H89 [38]). In line with this finding, cAMP-activated guanine nucleotide exchange factor 2 (Epac2, Figure 2) was also translocated by exenatide in human atrial preparations [38]. This translocation of Epac2 might contribute to the positive inotropic effect of exenatide [67]. Phospholamban phosphorylation would indicate that exenatide acts via increased uptake of Ca^2+^ into the sarcoplasmic reticulum in human atrial preparations (Figure 1). Semaglutide (100 nM) reduced the amount of pentameric phospholamban in isolated human cardiac preparations [45]. This might also facilitate the uptake of Ca^2+^ into the sarcoplasmic reticulum, because then SERCA will be less inhibited by phospholamban (Figure 1). Exenatide led to a translocation in isolated atrial preparations of glucose transporter 1 (GLUT1) to the cell membrane and led to a rise of glucose transport into cardiomyocytes which may contribute to its positive inotropic effects [38].

In the failing human ventricle, semaglutide (cf. Section 9) might also use the following mechanism of action [68]: the so-called late sodium current I_Na_ is augmented in failing human ventricles compared to non-failing human ventricular cardiomyocytes. In ventricular cardiomyocytes from patients with reduced ejection fraction (heart failure with reduced ejection fraction, systolic heart failure, HFrEF), semaglutide reduced this pathologically augmented late sodium current I_Na_ [68]. In ventricular cardiomyocytes from patients with HFrEF, semaglutide increased cytosolic Ca^2+^ transients [68]. Furthermore, in contraction studies in human atrial preparations, it turned out that GLP-1R agonists that did not act via β-arrestin possibly led to a longer-lasting positive inotropic effect which may result from less β-arrestin-mediated desensitization in isolated human right atrial preparations [38].

## 3. Force in Human Muscle Strips (Figure 1)

In a seminal paper, mentioned in the previous section, exenatide (cf. Section 6) and GLP-1(7-36)amide (cf. Section 5) raised force of contraction in isolated atrial muscle strips from non-failing human heart [38]. These positive inotropic effects were GLP-1R mediated because they were antagonized by exendin(9-39), a truncated derivative of exenatide which is an antagonist of the GLP-1R (Figure 1 [38,69]). Such a positive inotropic effect of GLP-1R agonists was later confirmed for exenatide and extended to other selective GLP-1R agonists like liraglutide (cf. Section 8) and semaglutide (cf. Section 9), all of which increased force of contraction in isolated human atrial preparations [41,45]. The positive inotropic effects of exenatide, liraglutide and semaglutide were accentuated by pretreatment of atrial muscle preparations with cilostamide, a phosphodiesterase inhibitor, supporting a signal transduction via cAMP [41]. As mentioned above, in principle exenatide and GLP-1(7-36)amide could raise force of contraction in ventricular muscle strips from non-failing human hearts, but only in a minority of patients [38]. Interestingly, when another laboratory studied isolated ventricular preparations from failing human hearts, they noted, in the absence of cilostamide, a positive inotropic effect of semaglutide that was accompanied by and probably caused by an increase in cAMP levels [68]. Hence, endogenous GLP-1 and exogenous GLP-1R agonists can raise force of contraction in the human heart. These positive inotropic effects occurred at therapeutic concentrations of the studied GLP-1R agonists.

## 4. Expression of Cardiac GLP-1R (Table 4)

Animal studies: Using antibodies that had specificity for the mouse pancreas, but probably not necessarily for the cardiac GLP-1R, it was noted that GLP-1R was present in cardiomyocytes, endocardium and microvascular endothelium and coronary smooth muscle cells but not in cardiac fibroblasts from adult mice [70]. The measurement of GLP-1R might be hampered by technical problems. The main problem seems to lie in a lack of specificity for GLP-1R in the currently available antibodies (e.g., [8]). For instance, using a commercial antibody in mouse left ventricle, mouse right ventricle and mouse septum the expression of GLP-1R was much higher than in the mouse atrium [70]. However, in a subsequent paper, the same group using reverse transcription polymerase chain reception (RT-PCR) failed to detect GLP-1R in mouse ventricular adult cardiomyocytes but still detected the mRNA for GLP-1R in adult mouse atrial cardiomyocytes [67]. The latter data are supported by findings in GLP-1R-Cre mice crossed with fluorescent reporter mouse strains [71]. In these mice, fluorescence and thus supposedly the GLP-1R was found in the aorta and in the arteries of the adult mouse heart [71]. Of note, no fluorescence was detectable in adult mouse cardiac ventricle with the exception of smooth muscle cells within the ventricle [71]. From looking at the histology, one would say that, in the atrium, most of the fluorescence was present in the cardiomyocytes and some fluorescence was located within smooth muscle cells [71]. Only about 10–20% of the atrial cardiomyocytes expressed the GLP-1R (based on their fluorescence). These histological data were in agreement with RT-PCR data in the same paper [71]. They noted the highest expression of the mRNA for the GLP-1R in mouse atrial samples, less in mouse aortic samples and very little in the mouse ventricular samples [71]. Up to now, at least two GLP-1R knockout mice have been generated, in which one could investigate the inotropic or chronotropic role of the GLP-1R in isolated mouse cardiac preparations [72,73]. If the GLP-1R is indeed functionally expressed in the sinus node of the adult mouse heart then it is hard to understand why GLP-1, liraglutide and semaglutide do not exert a positive chronotropic effect in the mouse heart. Hence, one explanation for this discrepancy might be that the GLP-1R is present but does not couple with the ion currents in the sinus node of the mouse heart. The alternative explanation raised by another report it that in the mouse heart the GLP-1R is only present in endothelial cells and not in cardiomyocytes [74]. In isolated left atrium from adult wild-type mice and from rats in Western blots GLP-1R was expressed (based on the specificity of the antibody used: ab218532, Abcam); this expression of GLP-1R in mouse heart went up by daily injection of liraglutide in mice for 14 days [75]. Hence, in mouse and rat hearts the expression of the GLP-1R can be regulated, but it remains to be re-investigated in which cells (endothelial cells versus cardiomyocytes) these receptors exist and are altered in their expression.

Human studies: The expression of GLP-1R in the human heart is clinically relevant (Table 4). Others detected with an antibody (monoclonal antibody (mAb) 3F52) the protein for the GLP-1R in human atrial cardiomyocytes and human ventricular cardiomyocytes but not in endothelial cells or smooth muscle cells of human coronary arteries [76]. Surprisingly, they noted that GLP-1R agonists dilated coronary human arteries [76]. This is counterintuitive because GLP-1R is supposedly not expressed in any cells of human coronaries that could induce a vasodilatation [76]. The authors tried to explain this by interactions between cardiomyocytes and the capillaries surrounding them [76]. An alternative explanation would be that the expression of GLP-1R exists in human coronary arteries but simply is below the level of detection with the methods currently available. Interestingly, the expression and hence function of GLP-1R fell in heart failure [68]: in cardiac ventricular samples from patients with heart failure with reduced ejection fraction (HFrEF), in Western blots less GLP-1R was detected than in controls (rabbit polyclonal antibody, Thermo Fisher (Waltham, MA, USA), 26196-1-AP). The authors used as controls ventricular cardiac samples from patients with heart failure with reduced ejection fraction (HFrEF) [68]. This finding suggests the cardiac GLP-1R can be down-regulated in disease in humans.

GLP-1R is also located in fat cells that normally are present in the epicardium of the human heart (identified using mRNA and immunofluorescent measurement of the protein GLP-1: [77], Table 4). The amount of fat in the epicardium can be measured non-invasively with echocardiography. In this way, it is possible to monitor the efficacy of GLP-1R agonists in the human heart by measuring a reduction of this epicardial fat [78]: at least exenatide and liraglutide reduced fat in the epicardium of patients [78,79].

**Table 4:** GLP-1R of expression and function in the human heart.

Region	Cell Type	GLP-1R Protein	GLP-1R mRNA	Function	Reference
Atrium	Sinus node	+ ^1,2,3^	+ ^2^ + ^4^		1: [76] 2: [80] 3: [81] 4: [82]
Atrium	Endothelial cells	−			[76]
Atrium	Smooth muscle cells	−			[76]
Left atrium		−	+		[80]
Right atrium		− ^1^	+ ^2^	PIE of GLP-1(7-36) ^3^ PIE of exenatide ^3^ PIE of semaglutide ^4^ PIE of liraglutide ^4^ No PIE of GLP-1(9-36) ^3^	1: [80] 2: [76] 3: [38] 4: [41]
Right atrial cardiomyocytes		+			[38]
Right ventricle	Whole tissue		+ ^1,2^	PIE of GLP-1(7-36) (two from one patient)^1^ No PIE of GLP-1(7-36) ^1^ PIE of exenatide^1^ (two samples from one patient), No PIE of exenatide ^1^	1: [38] 2: [80]
Right ventricle	Cardiomyocytes		+		[38]
Left ventricle	Whole tissue	+			[68]
Left ventricle	Cardiomyocytes	+ ^1^	+ ^2,3,4^		1: [76] 2: [80] 3: [74] 4: [38]
Left ventricle	Endothelial cells	+ ^1^	+ ^2,3^ − ^3^		1: [76] 2: [80] 3: [74]
Epicardium	Fat cells		+		[78]

First column indicates the region or the cell type in the human heart. Second column indicates the cell type studied of the region mentioned in the first column. Third column indicates a possible detection of the GLP-1R at the protein level (+) or a lack of detection (−). Fourth column indicates whether the GLP-1R is detected at the messenger ribonucleotide (mRNA) level (+) or a lack of detection (−). Fifth column refers to function of the stimulation of the GLP-1R in this animal, organ, or cell. Positive chronotropic effect (PCE), positive inotropic effect (PIE), atrial natriuretic factor (ANF), L-type calcium ion channel (LTCC). (+) indicates an agonism of the GIPR. Last column indicates references on this drug.

## 5. Physiological Relevance of GLP-1 in the Heart (Figure 1 and Figure 2)

Animal studies: Of note, the sequence of GLP-1 is identical in all mammals tested like guinea pig, hamster, human, mouse, rat and ox [83]. This might facilitate the translation of animal studies to the clinic [83]. However, species differences seem to exist. Mouse and rat data seem to mimic the effect of GLP-1 in the human heart poorly, whereas the heart of large animals like pigs shows more similarities to the human heart in our context. Injection of lixisenatide (a stable derivative of exenatide, Section 6) or liraglutide (a stable derivative of GLP-1, Section 8) led to a rise in the cardiac beating rate in living mice. These effects were reversed by pretreatment with propranolol, an antagonist of β-adrenoceptors [84], suggesting a role of released noradrenaline from cardiac stores and/or an enhanced sympathetic outflow from the brain. Moreover, as mentioned above, GLP-1(7-36)amide did not change the heart rate in isolated adult mouse hearts, whereas the mRNA of GLP-1R was present in the right atrium and left atrium but absent in the ventricle of mice [84]. The firing rate of the sinus node cells in isolated right atrial preparations from adult mice was not altered by liraglutide [84]. These data concur that GLP-1 receptors are not functional in the mouse right atrium [84]. The positive chronotropic effects of GLP-1R agonists in living mice appear to result from central or peripheral stimulation of the sympathetic nervous system and not direct action on the sinus node [84]. It may have clinical implications that, in acute experiments, GLP-1 agonists diminished any damage to the heart from ischemia in wild-type mice but not in mice with constitutional knockout of GLP-1R [62]. These cardiac-protective effects in mice are therefore probably mediated by GLP-1R. Interestingly, these GLP-1Rs are not expressed in the cardiomyocytes in mice but in the endocardial endothelial cells of the mice [74]. GLP-1 is also synthesized in the brain by neurons in regions of the nucleus tractus solitarius [85]. Therefore, central effects of GLP-1 formed in the brain, or of GLP-1R-agonistic drugs on brain GLP-1R receptors, are possible. Such effects may augment the sympathetic outflow from the brain. Central effects may elevate the beating rate of the heart in mice (and possibly humans). One might argue that GLP-1Rs do not couple with force generation in the mouse because they are located in the endothelial cells. However, there are biochemical functions that couple with the GLP-1R in the mouse cardiomyocyte: liraglutide raised cAMP levels and enhanced ANF secretion in isolated adult mouse atrial cardiomyocytes [67]. Hence, one could argue that others used methods that were not sensitive enough to identify GLP-1R in the mouse atrial cardiomyocytes [74] or that liraglutide acts not only on GLP-1R but also on other targets to induce secretion of ANF [67]. Moreover, the same group found liraglutide to raise cAMP in mouse atrial cardiomyocytes, which is difficult to understand if GLP-1Rs are lacking in these cells. The authors argued that beneficial effects of liraglutide in cardiac ischemia in mice are mediated via cardiac endothelial cells [74]. This does not seem to be the case in human hearts. The same paper convincingly reported that the mRNA of the GLP-1R in the human heart is only detected in human cardiomyocytes and not in human endothelial cells [74]. These data would explain why exenatide can have a positive inotropic effect in the human atrium [38] as the mRNA for the GLP-1R is found in cardiomyocytes in the human atrium [38,74].

In mice with constitutive ablation of the GLP-1R the heart rate was lower than in WT [72]. This was interpreted as evidence that the GLP-1R by itself enhanced the heart rate possibly in the sinus node of WT [72]. This does not prove a role of GLP-1R in the mouse sinus node for the regulation of the heart beat [72]. A central mechanism might underlie this regulation of the heart beat by GLP-1R agonists. Indeed, in mice with cardio-specific knockdown of the GLP-1R the heart rate response to an injection of liraglutide was similar to that in wild-type control mice, casting further doubt on a direct role of GLP-1R in the sinus node to control the heart rate at least in mice [86]. In vivo, the beneficial cardiac effects of GLP-1R in mice might well result from their anti-inflammatory effect on the function of leukocytes and cardiac fibroblasts [87,88]. In a mouse model of heart failure with down-regulation of sarcoplasmic reticulum Ca^2+^-ATPase (SERCA), exenatide led to an up-regulation of SERCA [89]. After ischemia by occlusion of a coronary artery, a combination of exenatide and cilostamide enhanced the level of cAMP and the activity of the cAMP-dependent protein kinase in these hearts compared to non-ischemia control mouse hearts (six weeks of age) and adult cardiomyocytes [90]. The combinations of exenatide and cilostamide enhanced the phosphorylation state of CREB, AKT or ERK1/2 [90]. These findings are counterintuitive in two ways. It is not apparent how a rise in cAMP and cAMP-dependent protein kinase would come about if there were no GLP-1Rs in the mouse ventricle. One explanation is that the cAMP derives from the atria and not from the ventricles. This rise of cAMP could result from a compartment in the mouse atrial cells that do not couple with force like mouse atrial endothelial cells. This issue probably needs a re-investigation with additional methods.

As concerns the production and degradation of GLP-1 in the gut and the heart, it seems noteworthy that in many animal models (e.g., insulin secretion from pancreas) the initially formed “full length” GLP-1(1-37) was either inactive or much less active than its proteolytic fragment GLP-1(7-36)amide (Figure 2). Of note, in the isolated rat heart, GLP-1(7-36)amide was formed when given GLP-1(1-36) [91]. In the perfused mouse heart, infused GLP-1(7-36)amide was rapidly converted to GLP-1(9-36)amide. GLP-1(9-36)amide is practically inactive as an agonist of GLP-1R [91]. In isolated beating adult rat hearts, this GLP-1 (7-36)amide raised the activity of MAPK and this argues for a functionally active GLP-1R in the rat heart [92]. In isolated adult rat ventricular cardiomyocytes, stimulation of GLP-1R augmented the cAMP levels but reduced contractility [93]. This reduced contractility was explained by a reduction of the pH in the isolated ventricular rat cardiomyocytes [93]. The authors also measured a desensitization of the rat ventricular myofilaments to Ca^2+^ in the presence of GLP-1(7-36)amide and in this way they explained a negative inotropic effect of GLP-1(7-36)amide in the rat heart, which is clearly opposite to the findings in the human heart [45,68,93]. Please also note that others failed to detect the mRNA of GLP-1R in isolated rat ventricular cardiomyocytes (e.g., [84,94]). In isolated perfused rat hearts, conflicting functional results were reported on the role of GLP-1R. In one study, GLP-1(7-36)amide was given to isolated adult rat hearts combined with a protease inhibitor (valine pyrrolidide, to ensure the intactness of GLP-1(7-36)amide [95]). However, the authors noted no change in left ventricular pressure or heart rate in their rat Langendorff-perfused hearts [95]. Likewise, GLP-1 (0.5 nM, more specifically they used GLP-1(7-36)amide without a protease inhibitor) reduced the left ventricular pressure without changing beating rate in isolated spontaneously beating rat hearts [92]. In contrast, in a more recent paper, GLP-1 raised the beating rate in isolated adult rat right atrial preparations [96]. This discrepancy between rat studies might stem from methodological differences (isolated atrium versus perfused heart, age, strain).

In pigs, there is convincing evidence that GLP-1(7–36) amide can have a positive chronotropic effect via GLP-1R in the sinus node [97]. For instance, GLP-1(7–36) amide enhanced the beating rate of the porcine heart upon injection [97]. Moreover, the mRNA for GLP-1R is present in porcine sinus node cardiomyocytes [97]. Stimulation of sinus node cells in the pig exerted electrophysiological changes that can explain a positive chronotropic effect [97]. GLP-1(7–36) amide enhanced the phosphorylation state of phospholamban at serine 16 in the sinus node of the pig [97].

Human studies: In the human intestine after a meal there was a rise in total GLP-1, of which 80% corresponded to GLP-1(7-36) amide and 20% to GLP-1(7-37) [98]. That means it seems to be appropriate to study mainly GLP-1(7-36) amide in contraction experiments of the human heart, however, even usage of the minor component GLP-1(7-37) (carrying glycine at the C-terminus) carries clinical relevance. Both peptides are degraded with a similar half-life (1–6 min [83,99]) and thus may be employed similarly in experimental studies. As mentioned above, in living mammals, GLP-1(7-36)amide is degraded by proteases (mainly dipeptidyl peptidase IV) but is also eliminated via the kidneys. This dipeptidyl peptidase IV is not only present in enterocytes but also in endothelial cells in capillaries [2]. Hence, degradation of GLP-1(7-36)amide could occur in the capillaries of the human heart and probably be inhibited by an inhibitor of the activity of dipeptidyl peptidase IV like sitagliptin [100]. In patients with impaired renal function, which often occurs in heart failure patients, high plasma levels of GLP-1 were described, probably due to impaired renal elimination [83]. There is further evidence for a clinical regulation of concentration of GLP-1: in a recent clinical study, in cardiac ischemia GLP-1 levels fell [75]. GLP-1(1-36) itself has apparently not been studied with respect to force in human atrium preparations (HAPs). Maybe this was never tried because it was thought to be irrelevant. Of note, the affinity K_D_ value of GLP-1R of GLP-1(1-36) was about 1 µM whereas the affinity K_D_ value for GLP-1(7-36) was 4.1 nM [101]. Hence, GLP-1(7-36) is an example of a drug that acts only as an active metabolite; the metabolite is about 250-fold more potent than its precursor, GLP-1(1-39). However, there was a positive inotropic effect by GLP-1(7-39) in a study in all human atrial preparations tested but only in a few human ventricular preparations (non-failing hearts [38]. If one removes two additional amino acids from GLP-1(7-36)amide, then one obtains GLP(9-36)amide, which is ineffective to raise force in the isolated human atrium [38] (Figure 2). Somewhat surprisingly, the positive inotropic effect of GLP-1(7-36)amide only started at 60 nM (given the affinity of 4.1 nM, vide supra: [101]) whereas the positive inotropic effect of exenatide started at 6 nM in human atrial preparations [38]. A possible explanation for this apparent discrepancy may lie in pharmacokinetic differences between these compounds. The half-life of GLP-1(7-36)amide is in minutes in the plasma but is much longer for exenatide [102] (cf. Section 6). Hence, GLP-1(7-36)amide may be rapidly degraded in the heart. That would explain why higher concentrations of GLP-1(7-36)amide (60 nM) in comparison with exenatide (6 nM) are needed to observe a positive inotropic effect in the human heart [38].

## 6. Exenatide (Figure 1 and Figure 2, Table 1 and Table 2)

Animal studies: The affinity of exenatide was similar to that of GLP-1(7-37) for the recombinant human GLP-1R [42]. Exendin-4 (the naturally occurring form of exenatide) is a cloned protein. A transgenic mouse has been made with overexpression of exendin-4 [103]. One could regard this transgenic mouse as a model for high, persistent dose exenatide treatment in a patient. This long-term overexpression with exendin-4 led to compensatory changes in the physiology: the blood glucose levels in these mice were not different from those of wild-type mice, despite high blood levels of exendin-4 in the transgenic mice compared to control mice [103]. Interestingly, the transgene, exendin-4, was also expressed in the heart because a promoter with wide tissue activity was used [103]. These findings were interpreted as suggesting that continuous application of GLP-1R agonists is inferior in lowering of blood glucose levels compared to twice daily injection of exenatide which did reduce blood glucose levels in mice [103]. Thus, these data support the clinical value of daily injection of exenatide and would argue against long-term application of exenatide, for instance, from a depot in the body or via a pump (as used for insulin). Interestingly, in mouse left atrial preparations and in the mouse right atrial preparation in the absence and presence of the phosphodiesterase 4 inhibitor rolipram, exenatide failed to change force of contraction or beating rate [41].

Human studies: As mentioned above, exenatide at 6 nM elevated force of contraction to 116% that of the predrug value in isolated electrically driven atrial preparations from non-failing human hearts [38]. Thus GLP-1R stimulation is much less effective to raise force of contraction than stimulation of β-adrenoceptors with, e.g., isoprenaline. As detailed above, these positive inotropic effects of exenatide in human atrial preparations probably involve an increase in cAMP and activation of cAMP-dependent protein kinase [38]. Exenatide was more potent to raise force in the presence than in the absence of cilostamide, a phosphodiesterase 3 inhibitor, supporting the assumption that exenatide raises force of contraction via an elevation of cAMP levels in cardiomyocytes [41]. Exenatide raised the cardiac beating rate in patients at low concentrations where exenatide does not reduce blood pressure [104]. This would be consistent with the assumption that exenatide directly stimulates GLP-1R in the human sinus node [104]. When, for 24 h, blood pressure and beating rate were measured in the same patients, there was no correlation between a fall in blood pressure and beating rate [104]. This was regarded as evidence for a direct effect of GLP-1R agonists on GLP-1R in the human sinus node [104]. The clinical use of exenatide, the first GLP-1R agonist on the market, seems to be declining; novel drugs with longer half-life and drugs that can be taken orally have become available. For instance, in Germany (data from the year 2024) 1.3 million defined daily doses of exenatide were prescribed in comparison to 284 million defined daily doses for all GLP-1R agonists on the market (0.45% [105]).

## 7. Lixisenatide (Figure 3, Table 1 and Table 2)

Animal studies: As the name implies, lixisenatide is an artificial peptide modifying the sequence of exenatide. Indeed, the C-terminal proline in exenatide was exchanged for six amino acids (six lysines). This was done to prolong the half-life, allowing once daily injection. Interestingly, this sequence modification led to a molecule that was more potent than GLP-1 on GLP-1R (1.5 nM versus 5.5 nM, respectively [42,106]). Lixisenatide has the same binding affinity to GLP-1R as exenatide [27,107]. In contrast, lixisenatide is less potent than exenatide to raise cAMP in GLP-1R-transfected cells [107]. Lixisenatide did not change force of contraction or beating rate in isolated perfused mouse hearts [84]. It is hard to understand why, in isolated electrically driven ventricular cardiomyocytes from adult rats, 100 nM lixisenatide augmented the contractility (measured as fractional shortening of the cellular wall movement [94]). This effect was very large because lixisenatide was as effective as isoprenaline [94]. In contrast, GLP-1(7-36)amide itself failed to affect contractility in isolated electrically driven ventricular cardiomyocytes from adult rats, i.e., under the same conditions [94]. This is opposite to earlier reports where GLP-1(7-36)amide reduced contractility in isolated electrically driven adult ventricular rat cardiomyocytes by means of desensitization of myofilaments [93]. Like in rats, in isolated electrically driven ventricular cardiomyocytes from adult wild-type mice, 100 nM lixisenatide also raised the contractility [94]. In isolated electrically driven ventricular cardiomyocytes from adult mice with complete deletion of GLP-1R, 100 nM lixisenatide still enhanced contractility and was equieffective with isoprenaline. In a supplement data set, the authors also noted that exenatide and liraglutide augmented contractility in isolated adult mouse ventricular cardiomyocytes [94]. The authors suggested that lixenatide (and also liraglutide and exenatide) thus raised contractility independent of any involvement of GLP-1Rs in the mouse heart and probably also in the rat heart [94]. In the course of their studies on lixisenatide, the authors failed to detect the mRNA for GLP-1R in isolated rat ventricular cardiomyocytes [94]. This underscores the view that lixisenatide increased contractility in ventricular cardiomyocytes from rats by a yet unknown mechanism.

Human studies: It would be mechanistically and clinically interesting to know whether lixisenatide raises contractility in the human atrium or ventricle and if this occurred by GLP-1R stimulation and/or via additional mechanisms like in the rat and mouse (cf. preceding paragraph). But such studies are not yet available. Lixisenatide raised the beating rate in patients at therapeutic dosing, suggesting a direct or indirect stimulation of the human cardiac sinus node [104]. In some countries like Germany, lixisenatide is only available in a fixed combination with insulin glargine. The use of lixisenatide in Germany is rising (by 26%, data from the year 2024 and the previous year) and led to 7 million defined daily doses of lixisenatide in comparison to a total of 284 million defined daily doses for all GLP-1R agonists (2.5% [105]).

**Figure 3:**
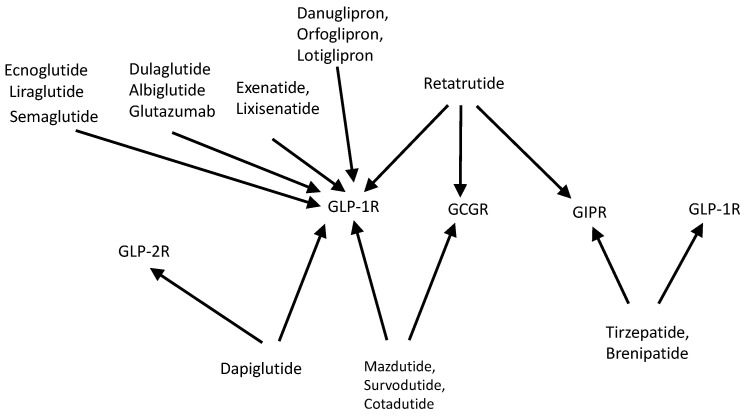
GLP-1R agonists or related drugs I (cf. Table 3). The drugs of interest are peptides or small organic molecules. The drugs stimulate the glucagon-like-protein-1 receptor (GLP-1R), the glucagon receptor (GCGR) or the glucose-dependent insulinotropic polypeptide (GIPR). Thus, danuglipron, lotiglipron and orforglipron are solely agonists of GLP-1R. The same holds true for the naturally occurring exenatide and its more stable derivative lixisenatide. Liraglutide, semaglutide and ecnoglutide closely resemble in their sequence the endogenous GLP-1. Likewise, dulaglutide and albiglutide are agonists of GLP-1R. Mazdutide, survodutide and cotadutide are agonists of both GLP-1R and GCGR. Tirzepatide and brenipatide are dual agonists of GCGR and GIPR. Retatrutide is a triple agonist of GLP-1R, GCGR and GIPR. Dapiglutide is a dual agonist of GLP-1R and the glucagon-like-protein-2 receptor (GLP-2R).

## 8. Liraglutide (Figure 3, Table 1 and Table 2)

Animal studies: Liraglutide was designed starting with the protein sequence of GLP-1 and modified such that the binding to albumin was enhanced [40]. This was achieved by coupling the fatty acid palmitate to the peptide backbone. Several fatty acids were compared and the fatty acid was chosen that did not reduce its affinity to GLP-1R [31]. The half-life of liraglutide is even longer because the injected liraglutide forms a heptamer near the injection site. This physicochemical feature is known in other peptides like mutant forms of insulin and prolonged their half-life [31]. This led to the possibility of once daily dosing in patients with type 2 diabetes. Atrial natriuretic factor (ANF) secretion was stimulated by liraglutide in mouse atrial cardiomyocytes (but not in mouse ventricular cardiomyocytes [67]). This is not a universal finding: others reported that liraglutide reduced the secretion of ANF in isolated adult rat atrium [75]. There may be methodological differences to explain this. Liraglutide elevated cAMP levels in neonatal ventricular mouse cardiomyocytes [108] and isolated adult mouse atrial cardiomyocytes [67]. As might be expected from these biochemical data, liraglutide (30 nM) alone failed to change left intraventricular pressure in isolated perfused adult mice hearts [108]. In contrast, in isolated adult mouse sinoatrial cardiomyocytes, 100 nM liraglutide raised the spontaneous beating rate [109]. Hence, again methodological differences may come into play.

Human studies: Liraglutide enhanced force of contraction in the isolated human atrium [41]. Liraglutide was more potent and effective to raise force in isolated human atrial preparations in the presence than in the absence of cilostamide, a phosphodiesterase 3 inhibitor, suggesting a cAMP-mediated effect [41]. The clinical use of liraglutide is rapidly declining at least according to German data (by 48% in the year 2024 compared to 2023). The use of liraglutide in Germany (data from the year 2024) led to 10.4 million defined daily doses in comparison to 284 million defined daily doses for all GLP-1R agonists on the market (3.7% [105]).

## 9. Semaglutide (Figure 3, Table 1 and Table 2)

Animal studies: Semaglutide was designed with the intention to be not immunogenic and to allow once weekly dosing of the drug. Hence, only one amino acid from the protein sequence of GLP-1 was exchanged with an artificial (not naturally occurring) amino acid (at position 8 of full length GLP-1) to impede the cleavage of GLP-1. Moreover, it was noticed that one could lengthen the half-life of this core peptide without compromising its potency by covalently linking it to a twenty-carbon-base diacid via a linker at amino acid 26 [31]. The binding of GLP-1(7-36)amide and semaglutide to GLP-1R in cryo-electron microscopy has been resolved and this revealed only slight structural differences [110,111]. Interestingly, with some galenic alterations it turned out that semaglutide can be made perorally active. However, dosage (and thus price) is high and food consumption has to be altered to improve bioavailability of semaglutide [31]: peroral semaglutide is given in the morning with 120 mL water before meals [112]. The bioavailability of semaglutide per os is reported to be as low as 1% [113,114,115]. Interestingly, in mouse left atrial preparations and in mouse right atrial preparations in the absence and presence of the phosphodiesterase 4 inhibitor, rolipram (used to amplify any cAMP formation), semaglutide failed to change force of contraction or beating rate as already mentioned for exenatide and liraglutide [41].

Human studies: Semaglutide enhanced force of contraction in the isolated human atrium more potently and effectively in the presence than in the absence of cilostamide, a phosphodiesterase 3 inhibitor, suggesting an involvement of cAMP [41,45]. Also, in ventricular muscle strips from patients with aortic stenosis or heart failure with reduced ejection fraction semaglutide (50 nM to 300 nM), it exerted a positive inotropic effect via GLP-1R [68]. This seems to have occurred in all tested patients and is at odds with a previous study that did not detect a positive inotropic effect in most isolated human ventricular muscle strips [38]. One may speculate that this is due to different experimental conditions because, e.g., Wallner et al. [38] only studied ventricular strips from non-failing human hearts and they used exenatide and not semaglutide to stimulate GLP-1R. The clinical use of semaglutide is rising fast (by 25% comparing the year 2024 to 2023 [105]). In Germany (data from the year 2024, from health insurance data) 129 million defined daily doses of semaglutide were prescribed in comparison to 284 million defined daily doses for all GLP-1R agonists (45% [105]).

## 10. Albiglutide (Figure 3, Table 1 and Table 2)

Animal studies: Albiglutide was developed with the intention to find a drug based on the protein sequence of endogenous GLP-1 but with a longer half-life than GLP-1. In albiglutide, as suggested by its name, albumin was covalently bound to a derivate of the protein sequence of GLP-1 [31]. More specifically, albiglutide is composed of a peptide starting at its amino-terminal end with two mutated copies of GLP-1. These mutations are single amino acid exchanges. At position eight, alanine is changed to glycine. Thereafter, the carboxyterminal end of albiglutide contains human albumin. The albumin part is supposed to impart further stability of albiglutide against proteases [21]. This manifests itself as a half-life for albiglutide of six days, allowing weekly injections [116]. This longer half-life is an advantage of albiglutide compared to the previously available drugs exenatide and liraglutide. Interestingly, in an animal model, albiglutide offered protection against cardiac damage by experimental ischemia and reperfusion, suggesting a protective role of albiglutide also in the ischemic human heart [117]. Exenatide was more potent than albiglutide to raise cAMP levels in a cell line that expressed GLP-1R [118]: while 0.1 nM exenatide started to raise cAMP levels about 30 nM of albiglutide was required [118]. Moreover, albiglutide has been tested in mouse models of diabetes type 2 and to treat obesity [118,119]. Hence, one would like to know whether or not albiglutide acts on force of contraction in the mouse heart.

Human studies: Albiglutide had the advantage against exentatide that it needed to be injected only once a week. Albiglutide got drug approval in the USA and Europe in 2013 and 2014, respectively (Table 1 [21]). Like other GLP-1R agonists, albiglutide improves the β-cell function in the pancreas of patients: the insulin levels were enhanced and the glucagon plasma concentrations were reduced [21]. In clinical studies, albiglutide reduced cardiovascular events [120,121]. Albiglutide enhanced the beating rate in patients which may result from direct cardiac effect or indirect compensatory effects [104]. However, because of strong competition from drugs with better efficacy, the sale of albiglutide fell and, supposedly for economic reasons, the distributor GlaxoSmithKline pulled albiglutide from the market worldwide in 2017 (Table 1).

## 11. Dulaglutide (Figure 3, Table 1 and Table 2)

Animal studies: The design of dulaglutide was probably based on a similar idea to that for albiglutide. Instead of fusing the core with albumin, a GLP-1-based peptide was connected with the Fc domain of human immunoglobulin G [31]. Dulaglutide, an approved drug (Table 1), was developed to activate GLP-1R in the pancreas [122]. Dulaglutide was designed in order to obtain a GLP-1R agonist that only needs to be given once a week [35]. To that end, the GLP-1 sequence was mutated to slow degradation by human proteases [35]. Direct fusion of GLP-1 and the immunoglobulin fragment greatly diminished receptor affinity [35]. Therefore, an array of small peptide linkers was tested and the best linker was used for dulaglutide. Nevertheless, the potency and the efficacy of dulaglutide to heighten cAMP in cell culture were lower than those of exenatide [35,123]. Dulaglutide did not affect force of contraction or beating rate in isolated mouse cardiac preparations [124].

Human studies: Dulaglutide reduced blood pressure and augmented heart rate in patients in a clinical study [125]. Dulaglutide exerted a positive inotropic effect in the isolated human atrium via GLP-1R [124]. Dulaglutide is often prescribed worldwide. The clinical use of dulaglutide in Germany (and probably also in other countries) is declining (by 18% comparing the year 2024 to 2023). Dulaglutide remains at present the most often prescribed GLP-1R agonist in many countries [105]. In Germany (data from the year 2025) 136 million defined daily doses of dulaglutide were prescribed in comparison to a total of 284 million defined daily doses for all GLP-1R agonists (49% [105]). However, dulaglutide prescription is losing ground compared to semaglutide (Germany: [105]).

## 12. Ecnoglutide (Figure 3, Table 1 and Table 2)

Animal studies: Ecnoglutide has a peptide backbone and contains a fatty acid (C18) coupled to amino acid 30 of the peptide sequence, which leads to a prolonged half-life. The core peptide is based on the sequence of GLP-1(7-36) and it contains a single mutation of the amino acid alanine 8 against valine. It was argued that the peptide, in contrast to liraglutide or semaglutide, contains only naturally occurring amino acids and hence the production will be cheaper and the price therefore lower for the customer and should be less likely to lead to allergic reactions [25]. Moreover, in contrast to liraglutide and semaglutide, ecnoglutide did not recruit β-arrestin and hence does not lead to internalization and GLP-1R inactivation and therefore a loss in efficacy. Ecnoglutide had an EC_50_ value of 0.025 nM (similar to semaglutide) on GLP-1R [36]. Only more than 10 µM ecnoglutide led to GLP-1R desensitization in cell-based assays [36]. Indeed, the efficacy of ecnoglutide was 60% of that of semaglutide to induce internalization in cell-based assay [36].

Human studies: The half-life in human healthy volunteers amounted to 124–138 h [36]. Ecnoglutide reduced body weight in patients when given by weekly injections. In contrast, tirzepatide shows more internalization than ecnoglutide and hence it has been speculated that effects of ecnoglutide in weight reduction might be more long lasting than those of tirzepatide [126]. In 2026 ecnoglutide was approved for treatment for type 2 diabetes in China (Table 1). Approval for weight reduction will probably be sought.

## 13. Glutazumab (Figure 3, Table 2)

Animal studies: Glutazumab comprises a protease-resistant variant of the protein sequence of GLP-1(7-36)amide that is covalently bound to the light chains of a humanized immunoglobulin G. Glutazumab activates GLP-1R in vitro [39]. Hence, it is similar to the older drugs dulaglutide and albiglutide in its structure [39]. It is assumed that both the antibody and the core peptide activate GLP-1R. Perhaps, therefore, glutazumab is more potent than GLP-1(7-36)amide alone to activate GLP-1R in vitro [39]. Glutazumab reduced body weight in mice [39].

Human studies: Glutazumab is currently undergoing clinical studies in China [127]. Glutazumab is thought to be entering the market in China in the near future.

## 14. Danuglipron, Orforglipron, Lotiglipron and CT-996 (Figure 3, Table 2)

Animal studies: The search for a small organic molecule that acts as an agonist of GLP-1R has been called the search for the “Holy Grail” in GLP-1R research [11]. One might say that the Holy Grail was found with danuglipron, lotiglipron and orforglipron. Danuglipron, lotiglipron and orforglipron are small organic molecules [34,44]. It has been argued that small molecules would not only reduce the price of drugs, making them available in poorer countries and for poorer parts of the population in general, but also would improve patient compliance. This is plausible because usually the synthesis of small organic compounds is done with equipment and supplies available since the 19th century. A further GLP-1R agonist is lotiglipron from Pfizer (patent: US00000010676465B220200609). Lotiglipron is a quite selective orally applied drug. It can be given once daily [43]. Lotiglipron promotes cAMP accumulation (EC_50_ value on GLP-1R of 27 nM) and stimulates β-arrestin 1/2 recruitment with an EC_50_ value of about 200 nM [128].

Human studies: The half-life of lotiglipron is 21–27 h in humans [129]. In a recent phase 2, prospective, randomized, double-blind, placebo-controlled, dose-ranging study, lotiglipron reduced blood glucose and reduced body weight in patients. The study was terminated because severe side effects were noted, such as transaminase elevation [43]. Both danuglipron and lotiglipron will probably not enter the market because in clinical studies liver toxicities were noted [130]. As far as we know, orforglipron is still being studied clinically and might soon enter the market [131] (Table 1).

## 15. Mazdutide (=IBI362, =LY3305677, Figure 3, Table 3)

Animal studies: Mazdutide was developed to improve on oxyntomodulin [49]. More specifically, mazdutide binds to human GLP-1 and GCGR less potently than oxyntomodulin with an affinity of about 29 nM and 18 nM, respectively [49]. Mazdutide was more potent to release insulin from murine pancreas cells than these numbers would predict, namely mazdutide stimulated release of insulin with an affinity of about 5 nM, suggesting a synergistic effect of the stimulation of GLP-1R and GCGR [49]. Mazdutide induced in mice a weight reduction mediated by both GLP-1R and GCGR [49]. In living mice, mazdutide reduced blood glucose levels, reduced hyperlipidemia and ameliorated serum triglycerides [49]. Mazdutide increased the beating rate in mouse atria via GCGR [50].

Human studies: Mazdutide reduced body weight and improved the metabolic profile in clinical studies [132,133]. Body weight fell by about 7% and blood glucose levels were reduced as was HbA_1c_ [133]. Please consider that mazdutide in some patients led to sinus tachycardia and other arrhythmias [133]. At the time of this writing, www.clinicaltrials.gov (accessed on 2 April 2026) includes at least seven completed or ongoing clinical studies. Moreover, mazdutide reduced systolic and diastolic blood pressure [134]. Mazdutide augmented force of contraction in isolated human atria via GCGR and GLP-1R [50]. Apparently, in China mazdutide has been filed for drug approval (Table 1).

## 16. Dapiglutide (Figure 3, Table 3)

Animal studies: Dapiglutide is also a dual agonist. More specifically, dapiglutide is an agonist of both GLP-1R and GLP-2R [48]. It is five times less potent than liraglutide on GLP-1R but has the same potency on GLP-2 receptors as native GLP-2. GLP-2 receptor agonism is beneficial in short bowel syndrome, a genetically coded disease in children. Like GLP-1, GLP-2 is also formed in special enterocytes called enteroendocrine cells [135]. First promising data in an animal model on gut function are available [48]. Clinical trials reported weight loss in humans. However, the developer (Zealand Pharma, Copenhagen, Denmark) stopped the clinical program to concentrate on survodutide (see below). In the adult rat perfused heart, GLP-2 alone and in the presence of isoprenaline reduced force of contraction [136]. This was unexpected for a drug that increases cAMP in GLP-2R-transfected cells [136].

Human studies: After surgical resection of parts of the gut in humans, a lack of GLP-2 can also occur which might benefit from dapiglutide [48]. One study has been completed (pharmacokinetic study) and another study is active but not recruiting (at www.clinicaltrials.gov; accessed on 2 April 2026). Dapiglutide will apparently not enter the clinic soon.

## 17. Survodutide (Figure 1 and Figure 3, Table 3)

Animal studies: Survodutide, like mazdutide, is a peptide based on the pharmacology of oxyntomodulin [53]. Chemical modifications of some amino acids in the sequence of survodutide lead to a much longer half-life (of about a week) for survodutide compared to the endogenous peptides and agonists of these receptors, namely, glucagon, GLP-1 or oxyntomodulin [53]. Survodutide can activate GCGR and GLP-1R in appropriately transfected cells in culture. Survodutide was more potent than oxyntomodulin and led to an augmentation in levels of cAMP in these cells with half-maximal stimulatory concentrations (EC_50_ values) of 0.015 nM for GLP-1R and 0.29 nM for GCGR [53]. Therefore, survodutide was 2.1-fold more potent on GLP-1R than oxyntomodulin [53]. In animal studies, survodutide was able to treat heart failure with preserved ejection fraction [137]. Interestingly, in mouse left atrial preparations in the absence and presence of the phosphodiesterase 4 inhibitor rolipram, survodutide failed to increase force of contraction [54]. However, survodutide increased the beating rate in mouse right atrial preparations via GCGR [54].

Human studies: In clinical trials, survodutide reduced obesity [138,139]. Survodutide has obtained a breakthrough designation from the FDA [32]. At concentrations that reduced body weight, in a clinical study survodutide increased heart rate and reduced systolic and diastolic blood pressure [139]. Survodutide was more potent and effective in the presence than in the absence of cilostamide, a phosphodiesterase 3 inhibitor, to raise force of contraction in the isolated human atrium via GCGR and GLP-1R [54].

## 18. Cotadutide (BI456906, Figure 3, Table 3)

Animal studies: Cotadutide (from AstraZeneca) is an investigational drug to treat obesity and type 2 diabetes. A crystal structure of cotadutide bound to GLP-1R and GCGR is available [140]. Like its analogues survodutide or mazdutide, cotadutide is a peptide based on the sequence of oxyntomodulin [47,53]. Cotadutide has been developed to treat type 2 diabetes, obesity and non-alcoholic steatohepatitis [47,53,141]. Indeed, cotadutide used glucagon receptors (GCGRs) and glucagon-like-peptide-1 receptors (GLP-1Rs) for its effects. Cotadutide lowers lipids and improves mitochondrial function via GCGR [141]. The peptide sequence of cotadutide contains parts of the sequence of glucagon and of glucagon-like-peptide-1 [47]. Chemical modifications in the sequence, namely, exchanges of amino acids 17, 20 and 24, led to less proteolysis and those of the epsilon-amino group of lysine 10 of palmitic acid via a gamma-glutamic acid spacer led to a higher affinity at receptors but also to longer half-life of cotadutide compared to glucagon or GLP-1 [47]. Cotadutide raised cAMP levels in cells transfected with human GLP-1R and GCGR with EC_50_ values of about 1 nM and 10 nM, respectively [142]. Thus, cotadutide is less potent than mazdutide or survodutide to raise cAMP in receptor-transfected cells. In these assays, cotadutide was about 100-fold less potent than glucagon on glucagon receptors and about 15-fold less potent than GLP-1 to raise cAMP in GLP-1R-transfected cultured cells [142].

Human studies: In clinical trials, cotadutide, like survodutide and mazdutide, reduced obesity and HbA_1c_ levels in type 2 diabetic patients. Like its competitors, survodutide and mazdutide, cotadutide also reduced body weight in non-diabetic obese patients [138,139]. There are at least nine completed or ongoing clinical studies (in www.clinicaltrials.gov: accessed on 2 April 2026). In a phase 1 study, cotadutide raised the beating rate in healthy human volunteers [143]. It is currently unclear, but it is conceivable that cotadutide may enter the market.

## 19. Tirzepatide (Figure 1 and Figure 3, Table 1 and Table 3)

Animal studies: Tirzepatide exhibits a modified protein sequence based on the sequences for GLP-1 and GIP [33]. Tirzepatide has a sequence of thirty-nine amino acids. Tirzepatide contains non-conventional amino acid residues at positions two and thirteen (α-amino isobutyric acids). The C-terminus of tirzepatide is amidated [144]. Tirzepatide at position 20, lysine, covalently binds a fatty acid moiety. This modification prolongs the half-life of tirzepatide further [145]. Tirzepatide acts as an agonist with high affinity (similar to endogenous GIP) at GIPR and binds with lower affinity to GLP-1R than endogenous GLP-1 [144,146]. Tirzepatide has an about forty-fold higher affinity for GIPR than for GLP-1R [144].

Human studies: Tirzepatide is an approved drug that is used to treat type 2 diabetes [55,147]. Tirzepatide reduced blood glucose levels. Tirzepatide led to weight loss in humans [33,55]. Tirzepatide was successful to treat chronic diastolic heart failure in patients [148] but may also be tried in sepsis or adriamycin-induced heart failure [149,150]. The effect of tirzepatide was more potent and effective in the presence than in the absence of cilostamide, a phosphodiesterase 3 inhibitor, to raise force of contraction in the isolated human atrium [56]. As concerns the usage in patients, at least in Germany in 2024, 19.8 million defined daily doses of tirzepatide were prescribed compared to 248 million defined daily doses for selective GLP-1R agonists (7.9% [105]). This was the first year (2024) tirzepatide was on the market. Therefore, tirzepatide was a successful new drug.

## 20. Brenipatide (Figure 3, Table 3)

Animal studies: Brenipatide is a unimolecular peptide (LY3537031). We failed to obtain publicly available preclinical data on brenipatide or LY3537031. It is only mentioned in a review [46]. Brenipatide is a dual agonist like tirzepatide [46]. Brenipatide stimulates GLP-1R and GIPR [46]. Somewhat surprisingly, brenipatide is tested to treat diseases such alcoholism, bipolar disorder and smoking withdrawal [151].

Human studies: Brenipatide is studied to treat bipolar disorders (NCT0728675), alcohol use disorders (NCT07219966) and major depressive disorders (NCT07412756). In a more expected fashion, brenipatide is also intended to treat obesity (NCT06606106) and liver disease (NCT07165002 at www.clinicaltrials.gov accessed on 2 April 2026). Brenipatide has the advantage of a longer half-life compared to tirzepatide and needs to be applied only once per month [46]. Hardly any clinical data on brenipatide are currently available. Clinical trials are apparently ongoing and, depending on their outcome, brenipatide could receive drug approval. It is unclear whether this drug will enter the market [46].

## 21. Retatrutide (Figure 1 and Figure 3, Table 3)

Animal studies: Retatrutide could activate GCGR, GIPR and GLP-1R in transfected cells in cell culture [51]. This activation raised cAMP levels in these cells [51]. In monkeys, retatrutide elevated heart rate and decreased blood pressure [30,51]. Interestingly, in mouse left atrial preparations in the absence and presence of a phosphodiesterase 4 inhibitor, rolipram, retatrutide failed to raise force of contraction. However, in isolated mouse right atrial preparations retatrutide augmented the beating rate and this effect was GCGR mediated [152]. These effects of retatrutide were mediated by GLP-1, GIPR and GCGR [152].

Human studies: Retatrutide was successful to treat diabetes type 2 diabetes and obesity [30,51]. In humans, retatrutide elevated heart rate and decreased blood pressure [30,51]. Retatrutide was more potent and effective in the presence of cilostamide, a phosphodiesterase 3 inhibitor, to raise force of contraction in the isolated human atrium [52]. These positive inotropic effects of retatrutide were mediated by GLP-1R, GIPR and GCGR [52]. Retatrutide will probably be approved in the near future (Table 1).

## 22. Summary

Our aim was to describe known or expected direct acute effects of GLP-1R agonists and closely related drugs on the isolated human heart. We would predict that all drugs that are agonists of GLP-1R, GIPR and/or GCGR would heighten force of contraction and conceivably raise the beating rate by a direct stimulation of the sinus node. Hence, one could test all drugs listed here as agonists of GLP-1R in human cardiac preparations. Moreover, as was already shown for retatrutide, tirzepatide or survodutide, it is expected that other di- and triagonists of GIPR and GCGR will have more potent and/or more effective contractile effects than pure GLP-1R agonists. There seems to be an immense interest of drug companies all over the world in developing such compounds (Table 1). Moreover, if a dual, triple or even quadruple agonists of GLP-1R and additional receptors that are expressed and are functional in the heart are produced, it may be useful to also test those drugs in human atrial preparations to predict clinical effects. One has to distinguish between acute effects and chronic cardiac effects of the abovementioned drugs. Both questions require further clinical studies for novel drugs. GLP-1R agonists gain additional indications such as the treatment of Parkinson’s disease, alcoholism or depression [9,11,12,153]. For these additional indications, it is important to know which cardiac side effects can occur with GLP-1R agonists. From the perspective of the patient, one would assume that new drugs should be taken per os, should possess a long half-life, should show no drug/drug interactions and should be free of detrimental cardiac side effects. One might note that some authors suggested from in vitro studies that the acute cardiac effects of, e.g., semaglutide might be beneficial to suppress arrhythmias and to sustain contractility in heart failure patients [68]. This should be tested in prospective clinical trials. It seems likely that GLP-1R agonists will be used widely in the future.

## Figures and Tables

**Table 1:** Approved GLP-1R agonists or coagonists or in drug combinations.

Drug	Approval Status	Side Effects	Improved Endpoints	Reference
Albiglutide (GlaxoSmithKline, London, UK)	2014 FDA, removed in 2018 for commercial reasons	Diarrhea ^1^, nausea, hypoglycemia, pancreatitis, injection site reaction, lung infections	Myocardial infarction ^2^	1: [21] 2: [22]
Cagrilintide + Semaglutide (Novo Nordisk, Copenhagen, Denmark)	2026 expected	Nausea ^1^, vomiting, diarrhea, abdominal pain, heartburn, injection site reactions		1: [23]
Dulaglutide (Eli Lilly, Indianapolis, IN, USA)	2014	Nausea ^1^, vomiting, diarrhea, injection site reactions	Stroke ^2^	1: [24] 2: [22]
Ecnoglutide (Sciwind Bioscience, Hangzhou, China)	2026	Nausea ^1^, diarrhea, lung infection, constipation, injection site reactions		1: [25]
Exendin-4 (=Exenatide) (AstraZeneca, Cambridge, UK)	2005	Nausea ^1^, vomiting, diarrhea, heartburn, injection site reactions, pancreatitis		1: [26]
Liraglutide (Novo Nordisk, Copenhagen, Denmark)	2010	Hypoglycemia ^1^, nausea, abdominal pain, angioedema, pancreatitis	Cardiovascular ^2^ death, myocardial infarction ^2^	1: [26] 2: [22]
Lixisenatide + Insulin Glargine (Sanofi, Paris, France)	2016	Nausea ^1^, vomiting, diarrhea, sore throat, hypoglycemia, injection site reactions		1: [27]
Mazdutide (Eli Lilly, Indianapolis, IN, USA)	2025 China	Nausea ^1^, vomiting, injection site reactions		1: [28]
Orforglipron (Eli Lilly, Indianapolis, IN, USA)	2026	Nausea ^1^, vomiting, diarrhea, constipation, hypoglycemia		1: [29]
Retatrutide (Eli Lilly, Indianapolis, IN, USA)	2026 expected	Nausea ^1^, vomiting, diarrhea, constipation, injection site reactions		1: [30]
Semaglutide (Novo Nordisk, Copenhagen, Denmark)	2017 FDA	Nausea ^1^, vomiting, diarrhea, abdominal pain, heartburn, injection site reactions	Cardiovascular death ^2^	1: [31] 2: [22]
Survodutide (Boehringer Ingelheim, Ingelheim, Germany)	Break-through designation 2024 FDA	Nausea ^1^, vomiting, constipation, cholelithiasis		1: [32]
Tirzepatide (Eli Lilly, Indianapolis, IN, USA)	2022 FDA	Nausea ^1^, vomiting, diarrhea, constipation, abdominal pain		1: [33]

Name of GLP-1 agonist and producer are in the first column Second column indicates whether the drug is approved or soon to be approved and in the third column possible side effects, all of which are summarized under the first reference. Fourth column indicates improved endpoints in clinical studies. Last column indicates references on this drug.

**Table 2:** GLP-1R agonists.

Drug	Section	Pure Peptide	Modified Peptide	Small Organic Molecule	PIE in HAP	Reference
Albiglutide	10		+		ND	[21]
Danuglipron	14			+	ND	[34]
Dulaglutide	11		+		ND	[35]
Ecnoglutide	12		+		ND	[36]
Exendin-4 (=Exenatide)	6	+ ^1^			+ ^2^	1: [37] 2: [38]
Glutazumab	13	+ and anti-body			ND	[39]
Liraglutide	8		+1		+ ^2^	1: [40] 2: [41]
Lixisenatide	7	+			ND	[42]
Lotiglipron	14	+		+	ND	[43]
Orforglipron	14			+	ND	[44]
Semaglutide	9		+1		+ ^2,3^	1: [31] 2: [41] 3: [45]

Name of GLP-1 agonist is in the first column. Second column indicates the section where the drug is discussed. Third column indicates whether the drug is a pure peptide. Fourth column indicates that there is a modified peptide. Fifth column indicates (+) whether the drug is a small organic molecule. Sixth column indicates the presence of a positive inotropic effect (PIE) in human right atrial preparation (HAP). Last column indicates references on this drug. PIE: positive inotropic effect in isolated human atrial preparation (HAP). ND: not determined.

**Table 3:** GLP-1R agonists and related drugs of interest with ancillary effects.

Drug	Section	GLP-1R	GIPR	GCGR	Variant	PIE in HAP	Reference
Brenipatide	20	+	+		Side chain attached	ND	[46]
Cotadutide	18	+		+		ND	[47]
Dapiglutide	16	+			GLP-2 Agonist	ND	[48]
Mazdutide	15	+ ^1^		+ ^1^		+ ^2^	1: [49] 2: [50]
Retatrutide	21	+ ^1^	+ ^1^	+ ^1^		+ ^2^	1: [51] 2: [52]
Survodutide	17	+ ^1^		+ ^1^		+ ^2^	1: [53] 2: [54]
Tirzepatide	19	+ ^1^	+ ^1^			+ ^2^	1: [55] 2: [56]

Name of GLP-1R agonist or related drug in the first column. Second column indicates section where the drug is discussed. Third column indicates the agonism with GLP-1R (+). Fourth column indicates whether the drug is an agonist (+) of the GIPR. Fifth column indicates a possible agonism (+) with the GCGR. Sixth column indicates a positive inotropic effect (PIE) in isolated human atrial preparation (HAP). Seventh column indicates references on this drug. ND: not determined.

## Data Availability

The data of this study are available from the corresponding author upon reasonable request.

## Abbreviations

AKT	protein kinase B
ANF	atrial natriuretic factor
cAMP	3’,5’-cyclic adenosine monophosphate
CREB	cAMP response element-binding protein
ERK1/2	extracellular signal-regulated kinases 1 and 2
EC_50_-values	molar concentration with 50% of the maximum response
EPAC	exchange factor directly activated by cAMP
G-protein	guanosine-triphosphate-binding proteins (G-proteins)
Gs	stimulatory G-protein
GCGR	glucagon receptor
GIPR	gastric inhibitory peptide
GIPR	gastric inhibitory peptide receptor
GLP-1	glucagon-like-peptide-1
GLP-1R	glucagon-like-peptide-1 receptor
GLP-2	glucagon-like-peptide-2
GLP-2R	glucagon-like-peptide-2 receptor
MAPK	Mitogen-activated protein kinase
mRNA	messenger ribonucleic acid
PKC	protein kinase C
PI-3K	phosphoinositide kinase 3
PLC	phospholipase C
SERCA	sarcoplasmic reticulum calcium ATPase
